# Bibliographical Notices

**Published:** 1844-11-02

**Authors:** 


### BIBLIOGRAPHICAL NOTICES.

*A Dictionary of Practical Medicine ; comprising general Pathology, the Nature and Treatment of Diseases, Morbid Structures, &c.* By James Copland, M. D., F. R. S. *Edited, with additions,* by Charles A. Lee, M. D. New York : Henry G. Langley, 1844.

We have already expressed our opinion of the Dictionary of Dr. Copland, in a notice of various Medical Dictionaries contained in the Examiner of the 27th of July last, and cannot do better than repeat the language then employed : " The '*Dictionary of Practical Medicine,*' by Dr. Copland, is in every respect an extraordinary work ; wonderful to be executed wholly by one man. No single article can be referred to without the reader being astonished at the extent of information, and the classifying and condensing powers of the author." With this impression of its merits, we congratulate the profession on the prospect of its re-publication in this country, and at a price greatly below that of the London edition.

The work is announced to appear in twenty "*parts*," to be issued monthly, until completed. That the editor and publisher will faithfully perform their part we have no reason to doubt; but whether they shall be able to accomplish the task within the period proposed, will depend upon the success of the author in completing the work. Judging from the past, we have great apprehensions that a far longer time will elapse.

The delay, which has already occurred since the work was commenced, requires that it shall be carefully revised, to bring the earlier portions of it up to the present state of the science. For the performance of this task, the publisher has been fortunate in obtaining the services of a gentleman whose varied learning and industrious habits well qualify him for the undertaking. The number before us bears evidence of the judgment and care with which he has supplied the deficiencies in the text, and especially in relation to what may be called *American Medicine.* Part I. treats of Abdomen, Abortion, Abscess, Absorption, Acne, Adhesions, Adipose tissue, After-pains, Age, Amaurosis, Angina pectoris, Antipathy, Aorta, Apoplexy, Appetite, Arteries, Arts and Employments, Asphyxy, Asthma; occupying 144 pages of closely printed matter, in double column.

*The Diseases of Females : including those of Pregnancy and Childbed.* By Fleetwood Churchill, M. D., Author of "the Theory and Practice of Midwifery," Licentiate of the King and Queen's College of Physicians in Ireland—Physician to the Western Lying-in Hospital, and to the Adelaide Hospital—and Lecturer on Midwifery, and the Diseases of Women and Children, &c. &c. *Third American edition with Illustrations. With Notes,* by Robert M. Huston, M. D., &c. 8vo. pp. 571. Philadelphia : Lea and Blanchard. 1844

Of this publication, for obvious reasons, we have little to say. The writings of Dr. Churchill are too well known, both in Europe and America, to require any extensive notice or analysis. The present work has already reached a fifth edition, in about as many years—two in Europe, and now a third in this country. In the last English edition of the first part, or that which treats of the diseases of the non-pregnant female, the author has corrected some errors contained in the former edition, inserted many additional references to complete the literary history of each subject, and introduced some additional illustrations. These improvements are all contained in the present publication.

*Ligature of the Carotid.*—Mr. Greville Jones narrates a case where ligature of the carotid was practised, and was followed by slight cerebral disturbance, with paralysis of sensation and motion in the left side of the body. Sensation was restored entirely, and motion partially, by appropriate treatment.—*Med. Times.*

